# Role of 5-HT_3_ Receptor on Food Intake in Fed and Fasted Mice

**DOI:** 10.1371/journal.pone.0121473

**Published:** 2015-03-19

**Authors:** Bingjin Li, Dongyuan Shao, Yungang Luo, Pu Wang, Changhong Liu, Xingyi Zhang, Ranji Cui

**Affiliations:** 1 Jilin provincial key laboratory on molecular and chemical genetic, Second hospital of Jilin University, Changchun, 130024, China; 2 National Engineering Laboratory for Druggable Gene and Protein Screening, Northeast Normal University, Changchun, 130024, China; Hosptial Infantil Universitario Niño Jesús, CIBEROBN, SPAIN

## Abstract

**Background:**

Many studies have shown that 5-hydroxytryptamine (5-HT) receptor subtypes are involved in the regulation of feeding behavior. However, the relative contribution of 5-HT_3_ receptor remains unclear. The present study was aimed to investigate the role of 5-HT_3_ receptor in control of feeding behavior in fed and fasted mice.

**Methodology/Principal Findings:**

Food intake and expression of c-Fos, tyrosine hydroxylase (TH), proopiomelanocortin (POMC) and 5-HT in the brain were examined after acute treatment with 5-HT_3_ receptor agonist SR-57227 alone or in combination with 5-HT_3_ receptor antagonist ondansetron. Food intake was significantly inhibited within 3 h after acute treatment with SR 57227 in fasted mice but not fed mice, and this inhibition was blocked by ondansetron. Immunohistochemical study revealed that fasting-induced c-Fos expression was further enhanced by SR 57227 in the brainstem and the hypothalamus, and this enhancement was also blocked by ondansetron. Furthermore, the fasting-induced downregulation of POMC expression in the hypothalamus and the TH expression in the brain stem was blocked by SR 57227 in the fasted mice, and this effect of SR 57227 was also antagonized by ondansetron.

**Conclusion/Significance:**

Taken together, our findings suggest that the effect of SR 57227 on the control of feeding behavior in fasted mice may be, at least partially, related to the c-Fos expression in hypothalamus and brain stem, as well as POMC system in the hypothalamus and the TH system in the brain stem.

## Introduction

Over past decades a negative relationship between brain serotonin (5-HT) and food intake has been reported [1–3]. Augmentation of brain 5-HT inhibits food intake while depletion of brain 5-HT promotes hyperphagia and weight gain [2, 3]. So far many 5-HT receptor subtypes have been identified [3, 4]. Some 5-HT receptor subtypes including 5-HT_1A_ receptors, 5-HT_1B_ receptors, 5-HT_2A_ receptors and 5-HT_2C_ receptors appear to play an important role in feeding behavior [2, 3, 5]. However, the relative contribution of 5-HT_3_ receptor remains unclear.

Recently, many studies have shown that 5-HT_3_ receptor is another important 5-HT subtype for modulating feeding behavior [1, 6–8]. Activation of 5-HT_3_ receptor affects many feeding-related neurons, including proopiomelanocortin (POMC) neuron [6], catecholamine neuron [1], AgRP neuron [7] and neuropeptide Y (NPY) neuron [8]. The 5-HT_3_ receptor also plays an important role in interacting with other feeding related peptide-mediated signaling. For example, a selective 5-HT_3_ receptor antagonist ondansetron attenuated cholecystokinin (CCK) induced satiety and c-Fos in the dorsal medulla [9]. Furthermore, systemic administration of 5-HT reduced sucrose intake in a dose-dependent manner via 5-HT_3_ receptor [10], whereas the selective blockade of 5-HT_3_ receptor decreased satiation [11–13]. The cross-talk between 5-HT_3_ receptor, CCK and NPY systems has been also evidenced in human [8]. These results suggest that activation of the 5-HT_3_ receptor may be implicated in the inhibitory modulation of feeding behavior. On the other hand, Sugimoto et al. (2006) also reported that the peripheral 5-HT_3_ receptor agonist, 2-methyl-5-HT, has no effect on food intake in rat [14]. Furthermore, suppression of food intake by 263 mM Polycose was equally attenuated by ondansetron administered with a does of 1.0, 2.0, and 5.0 mg/kg; whereas ondansetron alone did not affect food intake [12]. In addition, ondansetron reduced palatable food consumption in fed rats but not fasted rats [15]. The above described data indicate that the influence of the 5-HT3 receptor in feeding behavior remains controversial to a great extent. Therefore, the present study was aimed to investigate the specific role of the 5-HT_3_ receptor in the fed and fast state.

It has been shown that several regions of the hypothalamus including the paraventricular nucleus of hypothalamus (PVN), dorsomedial hypothalamus (DMH), ventromedial hypothalamus (VMH), arcuate nucleus (ARC) and lateral hypothalamic area (LHA) are involved in food intake and energy homeostasis. ARC and LHA are two particularly important hypothalamic nuclei which regulate feeding behavior. Mechanistically, fasting-induced c-Fos is reversed by refeeding in these brain regions [16–20]. In addition, neurons sensitive to feeding-related signals have also been identified outside the hypothalamus, particularly within dorsal vagal complex (DVC) in brainstem [1, 21, 22]. The DVC is comprised of the nucleus of the solitary tract (NTS), the dorsal motor nucleus of the vagus (DMV) and the area postrema (AP). Therefore, brain stem and hypothalamus are widely investigated in feeding-related studies, and extensive reciprocal neuronal projections exist between brainstem and hypothalamic feeding circuits to provide an alternative pathway through which circulating satiety factors can communicate with the hypothalamus [1, 16–23]. 5-HT systems in the body mediate nutritional input and the drive to feed [24], and multiple subtypes of 5-HT receptors are involved in the feeding behavior via POMC neuron [5]. In addition, energy restriction reduced tyrosine hydroxylase (TH) mRNA expression in the several brain regions compared to controls [25]. Therefore, it is intriguing that whether 5-HT_3_ receptor plays a key role in coordinating POMC, catecholamine and 5-HT-mediated actions for the control of feeding behavior under fed and fasted state.

Therefore, in the present study, we investigated the role of 5-HT_3_ receptor in the control of feeding behavior in fed and fasted mice as well as the underlying mechanisms. We found that activation of 5-HT_3_ receptor exerted an inhibitory effect on feeding behavior in fasted mice, which may be, at least partially, related to the c-Fos expression in hypothalamus and brain stem, as well as the POMC system in the hypothalamus and the TH system in the brain.

## Methods and Materials

### Animals

Imprinting Control Region (ICR) strain male mice (Age, 5-week-old; body weight, 20 ± 2 g) were purchased from Jilin University. Each mouse was housed in an individual cage (25.5 × 15 × 14 cm) and left to acclimatize to the laboratory conditions for 5 days, during which time they had *ad-libitum* access to standard food and water. The mice were maintained in standard laboratory conditions: 23 ± 1°C and 12 h light/dark cycle (lights on/off at 6:00 a.m/6:00 p.m).

### Drugs

SR 57227 hydrochloride and ondansetron hydrochloride were used (Tocris Cookson Ltd, Bristol, UK). All the drugs were dissolved in saline. Doses of the SR 57227 and ondansetron were chosen based on previous reports [26, 27]. SR 57227 was injected 30 min after ondansetron administration.

### Ethics Statement

The study was conducted in accordance with the Guide for the Care and Use of Laboratory Animals published by National Institutes of Health and with the recommendations and approval of the Ethics Committee on Animal Experiments of the Northeast Normal University. All efforts were made to minimize suffering.

### Feeding behavior

In behavioral studies mice were deprived of food for 18 h as fasted group [22, 28]. Each animal was injected intraperitoneally (i.p) with SR 57227 or ondansetron according to a counterbalanced and blinded design. The cumulative food intakes were recorded at 1, 3 and 6 h. Mice were fasted for 18 h from 3:00 p.m to 9:00 a.m as reported previously [22, 28], and all food intake test started from 9:00 a.m to 3:00 p.m.

### Western blot analysis for POMC and TH

Mice were sacrificed by decapitation. The brain was quickly dissected out and placed on ice. The hypothalamus and brain stem were removed and quickly frozen to-80°C until later use.

The extracted hypothalamus and brain stem tissues were weighed and homogenized in lysis buffer [137 mM NaCl, 20 mM TRIS, 1% NP40, 10% glycerol, 1 mM phenylmethylsulfonyl fluoride (PMSF)], 10 μg/ml aprotinin, 1 μg/ml leupeptin, 0.5 mM sodium vanadate, 0.5mM sodium flouride) on ice. The homogenates were then centrifuged at 10,000 rpm, 4°C for 20 min. The supernatant was collected into new tubes and stored at −80°C until use.

Western blot analysis was performed according to our previous stuides [28, 29]. The samples were resolved on 10% SDS-PAGE and transferred to a polyvinylidene difluoride (PVDF) membrane by electroblotting. TH, POMC and β-actin were immunostained with TH (rabbit polyclonal antibody AB152 diluted 1:2000), POMC [Goat polyclonal:ab32893 (3:1000)] and β-actin [mouse monoclonol; KM9001 (1:8000)]. After several washes with TBST buffer, the membranes were incubated with the respective peroxidase labeled secondary antibodies (1:1500 for TH, 1:5000 for POMC and 1:8000 forβ-actin). Specific bands were quantified densitometrycally and the ratio between the intensity of POMC or TH and beta-actin from the same homogenate was calculated.

### C-Fos immunohistochemistry

All mice were first anesthetized with chloral hydrate (400 mg/kg, i.p.), and then decapitated 2 h after SR 57227 injection as reported previously [30]. Brains were removed, immediately frozen in and stored at −80°C. Immunohistochemistry of c-Fos was carried out as described previously [30]. Serial coronal sections from forebrain to brainstem, 30μm thick, were cut in a cryostat. Free-floating sections were rinsed in 0.05 M phosphate buffered saline (PBS; pH 7.4) and then incubated with 0.6% hydrogen peroxide in PBS to remove endogenous peroxidase activity. After rinsing again in PBS, the sections were incubated with primary antibody (1:1000 dilution in PBS containing 0.3% Triton X-100, 0.05% sodium azide, and 2% normal goat serum) for 72 h at 4°C. The c-Fos antibody (Santa Cruz Biotechnology, #sc-52) is a rabbit polyclonal antibody raised against a peptide mapping at the amino terminus of human c-Fos p62. The sections were than rinsed and incubated with a secondary antibody [biotinylated goat anti-rabbit IgG (Vector Laboratories, Burlingame, CA) 1:400 dilution in PBS with 0.3% Triton X-100] for 75 min at room temperature. After being rinsed, the sections were transferred into PBS containing 0.4% avidin-biotinylated horseradish peroxidase complex (Vector Laboratories, Burlingame, CA) for another 75 min. Following successive wash in PBS and 0.2 M sodium acetate buffer (pH 6.0), the reaction product was visualized using a glucose oxidase-diaminobenzidine-nickel method [31]. The reaction was terminated through washing the sections in sodium acetate buffer. Thereafter, the sections were mounted onto chrome-alum-gelatin-coated slides from 0.05 M PBS. After being air-dried, the sections were counted with neutral red, dehydrated through a graded alcohol series, cleared in xylene and finally cover slipped.

The neurons positive for c-Fos were identified by their characteristic dense brown nuclear staining within hypothalamus and brain stem under a light microscope. The investigator counting the c-Fos cells was blind to experimental conditions. The counting was performed on 4–8 sections per region as described previously [30]. Locations in the brain were confirmed by staining and reference to the primary literature and a mouse brain atlas [32].

### Corticosterone, ACTH and 5-HT analyses

Mice were decapitated individually and trunk blood was collected in plastic tubes. The tubes were left at 4°C overnight to clot, and centrifuged at 4°C for 15 min at 3000 rpm to remove the red blood cells. The serum was then transferred to Eppendorf tubes and stored at −20°C until use. Because hormone levels exhibit a diurnal rhythm, blood samples were collected within the same time window (12:00 a.m). The serum concentrations of corticosterone, ACTH (adrenocorticotropic hormone) and 5-HT were quantified using enzyme-linked immunosorbent assay (ELISA). Mouse corticosterone (CORT) ELISA Kit (Cusabio, Wuhan, China), ACTH (Jiancheng, Nanjing, China) and mouse 5-hydroxytryptamine (5-HT) ELISA kits (Jiancheng, Nanjing, China) were used for quantification of serum corticosterone, ACTH and 5-HT respectively, according to the manufacturers’ manuals and our previous report [28]. Briefly, a standard curve was obtained from the optical density of the standards, from which the concentrations of the samples were calculated.

### Data analysis

The data are presented as mean ± S.E.M. The data were analyzed using one-way or two-way analysis of variance (ANOVA). When significant differences were obtained, post-hoc comparisons within logical sets of means were performed using Dunnett's test. *P* values less than 0.05 were considered as significant.

## Results

### Effect of SR 57227 on food intake in fed and fasting mice

**Fig 1:**
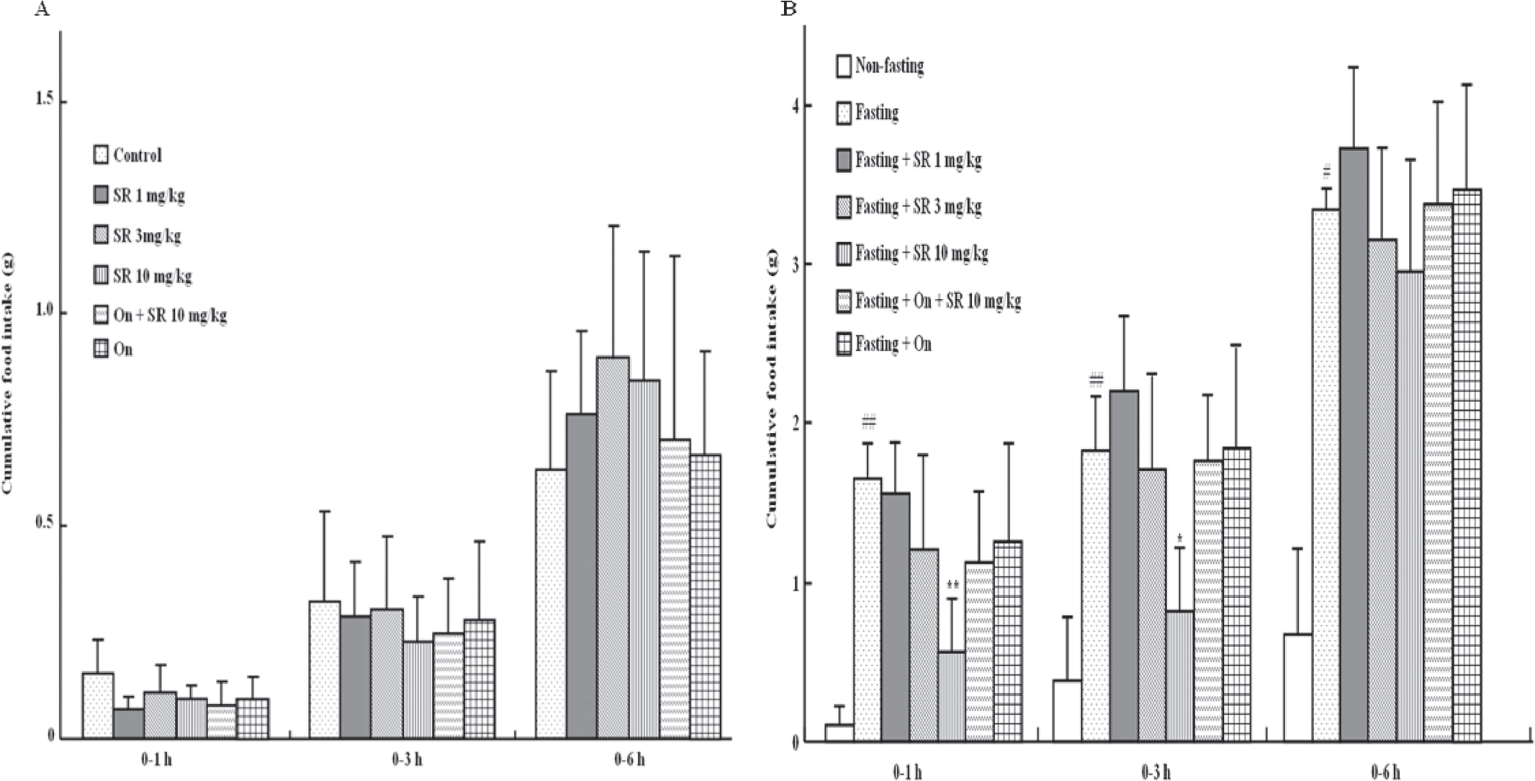
Effects of acute SR 57227 on cumulative food intake in fed and fasted mice. SR: SR 57227 (1, 3 and 10 mg/kg, i.p.); On: ondansetron (3 mg/kg, i.p.). Values are means ± SEM (n = 8–10). #, P < 0.05; ##, P < 0.01 vs. non-fasting group; *, P <0.05; **, P < 0.01 vs. fasting group.

In our first study all mice were divided into 6 groups: Control group (n = 10), SR 57227 groups (1, 3 and 10 mg/kg; n = 9), Ondansetron + SR57227 (10 mg/kg; n = 9), and Ondansetron group (n = 8). In fed mice two-way repeated-measures ANOVA showed that SR-57227 at the administrated doses (1, 3 and 10 mg/kg) had no effect on the cumulative food intake during 6 h period of test (F_(5, 53)_ = 1.40, P>0.05; Fig. 1A). In our second experiment all animals were divided into 7 groups: Non fasting group (n = 10), Fasting group (n = 9), Fasting + SR 57227 (1, 3 and 10 mg/kg, n = 9) groups, Fasting + Ondansetron + SR 57227 group (n = 8), Fasting + Ondansetron group (n = 9). Food deprivation for 18 h significantly enhanced the cumulative food intake during 1, 3 and 6 h periods of test (F_(5, 51)_ = 3.57, P<0.01; Fig. 1B). Furthermore, the cumulative food intakes during the first 1 h (P<0.01) and 3 h (P < 0.05; two-way ANOVA with Dunnett's test) but not 6 h were significantly inhibited by a high-dose of SR 57227 (10 mg/kg, i.p.) in the fasting mice (Fig. 1B). Particularly, the level of cumulative food intake in fasting mice treated with SR 57227 (10 mg/kg, i.p.) was returned to the level in corresponding non-fasting controls (Fig. 1B). The inhibitory effect of SR-57227 was blocked by ondansetron (Fig. 1B).

### Effect of SR57227 on c-Fos expression in the brain stem of fed and fasting mice

**Fig 2:**
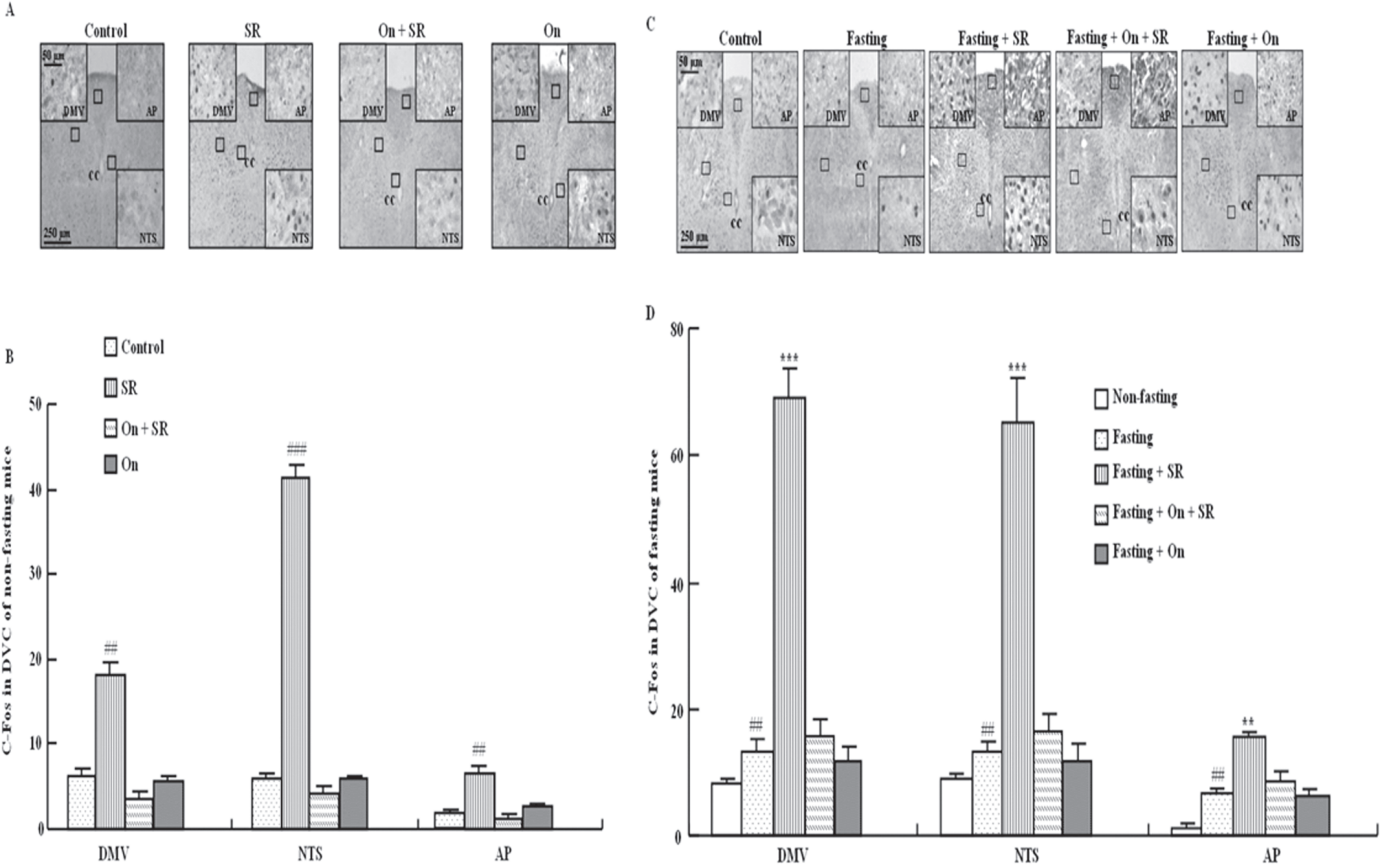
c-Fos expression in brain stem of fed and fasted mice. Typical photomicrographs of c-Fos expression in DVC of fed mice (A) and fasted mice (C); Mean number of c-Fos-positive cells in DVC of fed mice (B) and fasted mice (D). SR: SR 57227 (10 mg/kg, i.p.); On: ondansetron (3 mg/kg, i.p.). DVC: dorsal vagal complex; NTS: nucleus tractus solitarius; DMV: dorsal motor nucleus of the vagus; AP:area postrema; CC: central canal. Values are means ± SEM (n = 7–8). ##, P < 0.01; ###, P < 0.001 vs. non-fasting control group; **, P < 0.01; ***, P < 0.001 vs. fasting control group.

For the number of c-Fos-positive cells, one way ANOVA revealed that c-Fos expression was significantly increased by SR-57227 (10 mg/kg, i.p) in the DMV (F_(3, 28)_ = 55.82, P<0.01), NTS (F_(3, 28)_ = 88.0, P<0.01) and AP (F_(3, 28)_ = 49, P<0.01) of brain stem in fed mice (Fig. 2A and 2B), and these increases were blocked by ondansetron (Fig. 2A and 2B). Fasting for 18 h significantly increased c-Fos expression in the DMV (F_(3, 27)_ = 94.27, P<0.01), NTS (F_(3, 27)_ = 102.2, P<0.01) and AP (F_(3, 27)_ = 27.25, P<0.01) of brain stem in mice (Fig. 2C and 2D). Furthermore, the fasting-induced upregulation of c-Fos expression was enhanced by SR-57227, and these effects of SR-57227 were blocked by ondansetron (Fig. 2C and 2D).

### Effect of SR57227 on c-Fos expression in hypothalamus of fed and fasting mice

**Fig 3:**
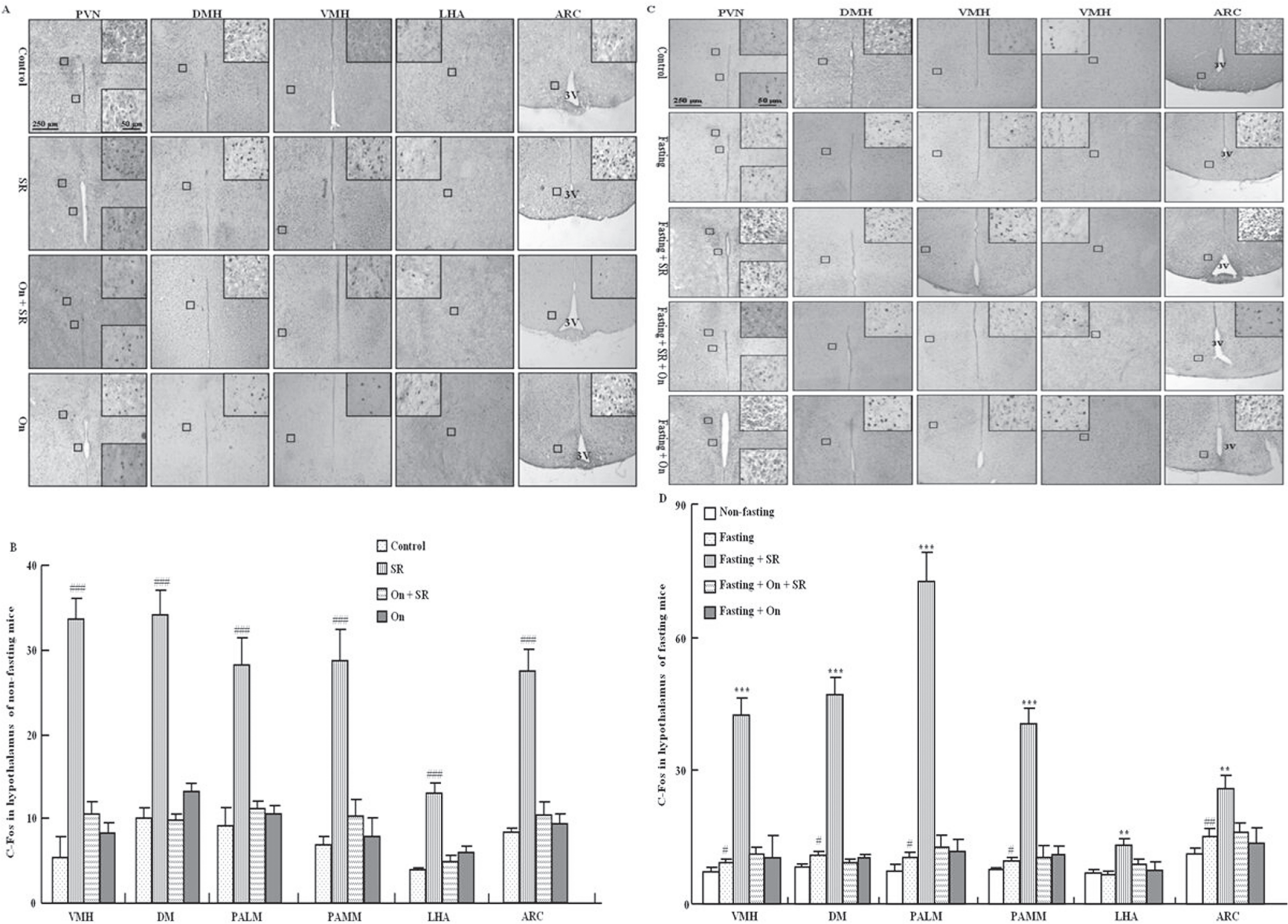
c-Fos expression in hypothalamus of fed and fasted mice. Typical photomicrographs of c-Fos expression in hypothalamus of fed mice (A) and fasted mice (C); Mean number of c-Fos-positive cells in hypothalamus of fed mice (B) and fasted mice (D). SR: SR 57227 (10 mg/kg, i.p.); On: ondansetron (3 mg/kg, i.p.). PVN: paraventricular nucleus of hypothalamus; DMH: dorsomedial hypothalamus; VMH: ventromedial hypothalamus; LHA: lateral hypothalamic area; PALM: paraventricular hypothalamic nucleus, lateral magnocellular part; PAMM: Paraventricular hypothalamic nucleus, medial magnocellular part; ARC: arcuate nuclei of the hypothalamus; 3V: third ventricle.Values are means ± SEM (n = 7–8). #, P < 0.05; ##, P < 0.01; ###, P < 0.001 vs. non-fasting control group; ** P < 0.01; ***, P < 0.001 vs. fasting control group.

As shown by one-way ANOVA, in fed mice SR-57227 significantly increased c-Fos expression in most areas of hypothalamus, including PVN, DMH, VMH, LHA and ARC (F_(3, 28)_ = 67.12, P<0.01) (Fig. 3A and 3B). Fasting increased c-Fos expression in these brain areas except LHA, and the c-Fos expressions were enhanced by SR-57227 (Fig. 3C and D). These enhancements of c-Fos expression induced by SR-57227 were attenuated by ondansetron in both fed and fasting mice (Fig. 3A–3D).

### Effect of SR57227 on TH level in hypothalamus and brain stem of fed and fasting mice

**Fig 4:**
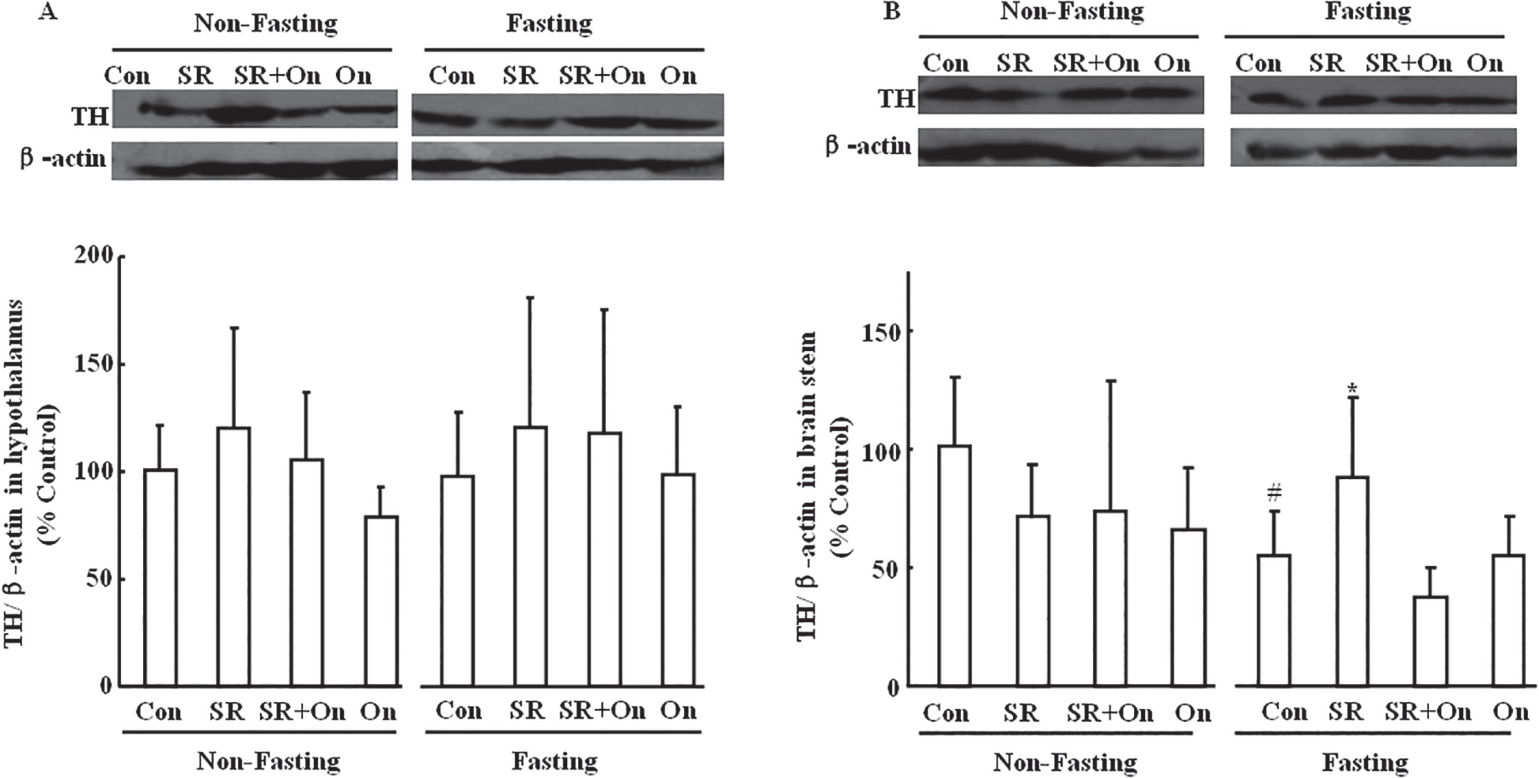
TH/β-actin ratio,normalized to control, in hypothalamus and bran stem of fed and fasted mice. Con: control group; SR: SR 57227 (10 mg/kg, i.p.); On: ondansetron (3 mg/kg, i.p.). TH: tyrosine hydroxylase. Values are means ± SEM (n = 8–10). #, P < 0.05 vs. non-fasting control group; *, P < 0.05 vs. fasting control group.

There was no significant change in the hypothalamic TH levels of fed and fasting mice after treatment with SR 57227 alone or in combination with ondansetron (Fig. 4A). However, the TH levels in the brain stem were significantly inhibited in fasting mice compared to the non-fasting controls (F_(7, 56)_ = 2.45, P = 0.03), and the inhibition of TH expression was attenuated by SR 57227 (F_(3, 27)_ = 3.76, P = 0.024) in the fasting mice (Fig. 4B). The effect of SR-57227 was antagonized by ondansetron (Fig. 4B).

### Effect of SR57227 on POMC expression in hypothalamus and brain stem of fed and fasting mice

**Fig 5:**
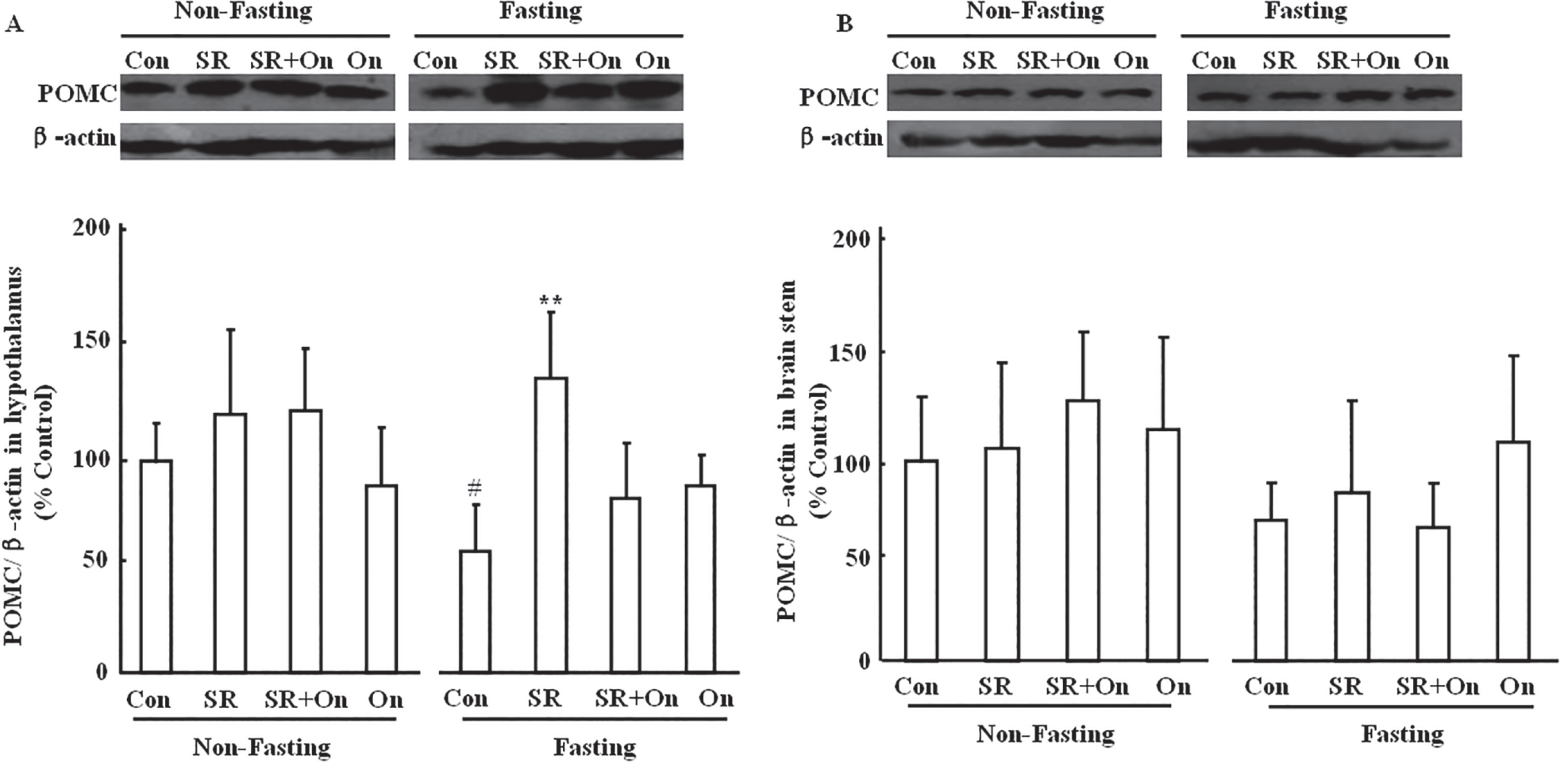
POMC/β-actin ratio,normalized to control, in hypothalamus and bran stem of fed and fasted mice. Con: control group; SR: SR 57227 (10 mg/kg, i.p.); On: ondansetron (3 mg/kg, i.p.). POMC: Pro-opiomelanocortin. Values are means ± SEM (n = 8–10). #, P < 0.05 vs. non-fasting control group; **, P < 0.01 vs. fasting control group.

There was no significant effect on the POMC protein expression in brain stem of fed and fasting mice after treatment with SR-57227 alone or in combination with ondansetron (Fig. 5A). However, hypothalamic POMC expression was inhibited in fasting mice compared to the non-fasting control (F_(7, 56)_ = 2.24, P = 0.04), and the inhibitory effect was suppressed by SR-57227 (Fig. 5B) (F_(3, 27)_ = 5.16, P = 0.0068). The inhibitory effect of SR-57227 was blocked by ondansetron.

### Effect of SR 57227 on 5-HT levels

**Fig 6:**
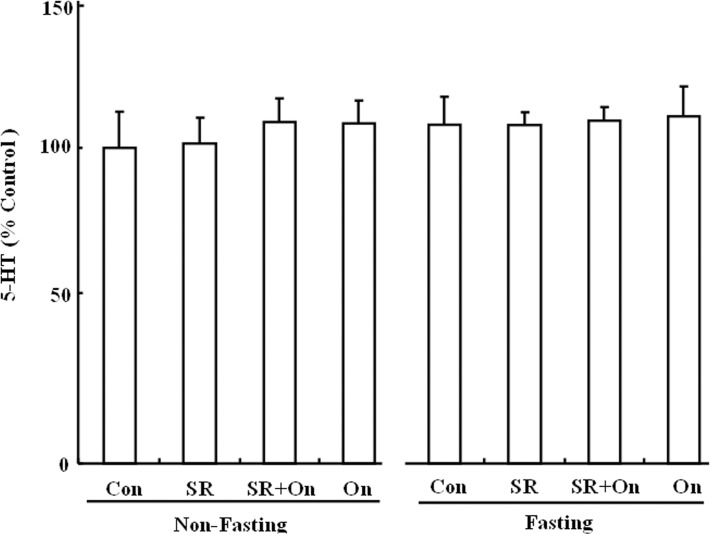
Serum 5-HT levels, normalized to control, in fed and fasted mice. Con: control group; SR: SR 57227 (10 mg/kg, i.p.); On: ondansetron (3 mg/kg, i.p.). Values are means ± SEM (n = 8–10).

5-HT levels of serum were examined in fed and fasted mice after treatment with SR 57227 alone or in combination with ondansetron. As shown by one-way ANOVA followed by Dunnett's test, there was no statistically significant difference (F_(7, 68)_ = 1.47, P = 0.19) (Fig. 6).

### Effect of SR 57227 on Corticosterone and ACTH levels

**Fig 7:**
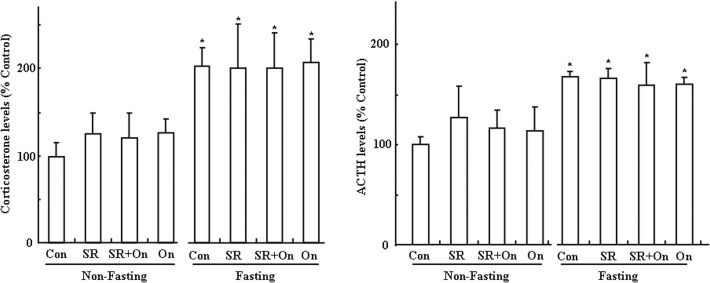
Corticosterone and ACTH levels, normalized to control, in fed and fasted mice. Con: control group; SR: SR 57227 (10 mg/kg, i.p.); On: ondansetron (3 mg/kg, i.p.). Values are means ± SEM (n = 6 or 7).*, P < 0.05 vs. non-fasting control group.

Corticosterone and ACTH levels were examined in fed and fasted mice after treatment with SR 57227 alone or in combination with ondansetron (Fig. 7). Corticosterone (F_(7, 52)_ = 2.765, P = 0.018) and ACTH levels (F_(7, 52)_ = 2.24, P = 0.048) were significantly increased by fasting. However, it was not affected by SR 57227 alone or in combination with ondansetron.

## Discussion

Most studies have shown that food intake after food deprivation is increased in rats and mice [33–34]. In the present study, cumulative food intake was significantly enhanced after 1, 3 or 6 h of fasting in mice, and the enhanced food intakes during the 1 and 3 h but not 6 h were suppressed by a 5-HT_3_ agonist SR57227. Particularly, the level of 3 h inhibitory effect of SR 57227 on the cumulative food intake in fasting mice treated with SR 57227 was returned to the corresponding non-fasting control level. Furthermore, the inhibitory effect of SR 57227 on fasting-induced food intake was blocked by a 5-HT_3_ antagonist ondansetron. Of note, it has been shown that 5-HT reduces sucrose intake in a dose-dependent manner via 5-HT_3_ receptor [10], while 5-HT_3_ receptor participates in the inhibition of food intake [12]. Therefore, these results demonstrate that activation of 5-HT_3_ receptor may be involved in the control of feeding behavior under fasting status. However, we did not find any significant change of food intake in fed mice after treatment with SR-57227 alone or in combination with ondansetron. Similarly, it has been reported that another 5-HT_3_ receptor agonist, 2-methyl-5-HT, has no effect on food intake in fed rats [14], and ondansetron alone did not affect food intake in fed rats [12]. These findings suggest that the 5-HT_3_ receptor may play different functional roles in regulating food intake under fed and fasting conditions.

Food deprivation increases neuronal activity (as measured by c-Fos immunoreactivity) in the feeding related brain sites [35]. A number of feeding related peptides, including CCK and ghrelin, require an intact vagal-brainstem-hypothalamic pathway to affect food intake. Therefore, to clarify the role of 5-HT_3_ receptors in regulating food intake in fed mice and fasted mice, we further investigated the effects of SR 57227 on c-Fos expression in the brain stem and the hypothalamus. SR 57227 increased c-Fos expression in most of brain stem and hypothalamus of mice. Furthermore, fasting-induced c-Fos expression was further enhanced by SR 57227 in hypothalamus (PVN, DMH, VMH and ARC) and brain stem (DMV, NTS and AP), and this enhancement was also blocked by ondansetron. In the present study, the effects of SR 57227 on c-Fos expression are non-specific under fasted and non-fasted mice. However, these data suggest that hypothalamus-brain stem circuitry may be, at least partially, responsible for the effects of SR 57227 on feeding behavior under fasted conditions. In general, directly acting agonists at 5-HT_1B_, 5-HT_2C_ or 5-HT_2A_ receptors decreased food intake, whereas stimulation of the 5-HT_1B_ and 5-HT_2C_ subtypes may probe physiological roles in feeding and satiety [36]. These data are inconsistent with our findings that activation of 5-HT_3_ receptor may inhibit feeding behavior under fasting condition. As we know, The 5-HT_3_ receptor is the only ligand-gated receptor, whereas all the other 5-HT receptor subtypes are G protein-coupled receptors, however, whether the inhibitory effect of 5-HT_3_ receptor on feeding behavior is also related to its structureneeds further investigation. In addition, another study showed that intravenous injection of 5-HT_3_ receptor antagonist granisetron blocked c-fos expression in all brain nuclei examined, although intracerebroventricular injection of granisetron alone had no effect, suggesting that 5-HT released from the stomach may activate 5-HT_3_ receptors located in the peripheral vagal afferent nerve terminals and then induce brain c-Fos expression. And c-Fos positive cells in the NTS were labeled with retrograde tracer fluorogold injected in the PVN, suggesting that neurons in the NTS activated by gastric distension project axons to the PVN [37]. Thus, whether a similar pathway was involved in the effect of SR 57227 needs to be further examined.

POMC and catecholamine systems play important roles in control of feeding behavior, the effect of 5-HT_3_ receptor activation on POMC and catecholamine levels in brain stem-hypothalamus pathway was examined in fed and fasted mice. Fasting significantly suppressed TH level in brain stem, and this effect was antagonized by SR 57227. Furthermore, this effect of SR 57227 was also reversed by ondansetron. TH is the entry enzyme into catecholamine biosynthesis and frequently used as a marker for catecholaminergic neurons [38]. Most recent report also showed that overnight food deprivation markedly attenuated hindbrain norepinephrine level in rats [39]. These findings suggest that TH in brain stem might thus exert some effect on the control of food intake in fasted mice via activation of 5-HT_3_ receptor. This finding also supported our previous report that TH neurons response to ghrelin is increased during fasting in brain stem, particularly in NTS [22]. In addition, TH levels in the hypothalamus were not signifcantly changed in fasted mice. Previously biochemical and behavioral studies support the role of hypothalamic catecholamine neurons in the regulation of food intake [16, 40, 41]. Food deprivation did not change hypothalamic norepinephrine level but increased dopamine level [42]. Another study also showed that 48 h food deprivation only decreased norepinephrine in ARC and ventromedial hypothalamus (VH), and dopamine in VH but not other brain sites of hypothalamus [43]. These different results suggest that fasting shows diverse effects on catecholamine system (norepinephrine, dopamine and epinephrine) in different hypothalamic sites. These findings may also explain that TH levels in the hypothalamus were not changed in fasted mice. On the other hand, POMC levels in hypothalamus were significantly reduced in fasted mice. Similarly both acute food deprivation and chronic food restriction decreased hypothalamic POMC gene expression [32–33]. Furthermore, the reduction of fasting-induced POMC level was reversed by SR 57227, and the effect of SR 57227 was blocked by ondansetron in fasted mice. These findings suggest that POMC in hypothalamus plays a more important role on control of food intake in fasted mice compared to that of brain stem. In contrast, there was a trend towards an overall decrease in the POMC level in brain stem of fasted mice but this was not significant.

Finally, 5-HT level was further investigated in fed and fasted mice after SR 57227 alone or in combination with ondansetron. But there were no statistically significant differences. Our previous report also showed that serum 5-HT level was not significantly changed after different time periods of fasting in mice [28]. These findings suggest that peripheral 5-HT may be not directly involved in the control of feeding behavior induced by fasting. In rodents, directly acting agonists at 5-HT_1B_, 5-HT_2C_ or 5-HT_2A_ receptors decreased food intake, whereas stimulation of the 5-HT_1B_ and 5-HT_2C_ subtypes may probe physiological roles in feeding and satiety [36]. However, whether these subtype receptors are also involved in the effect of SR 57227 on feeding behavior under fasting conditions need further examination. In addition, the 5-HT_3_ receptors are extensively expressed throughout the central and peripheral nervous systems, and mediate a variety of physiological functions [44]. On a cellular level, it has been shown that postsynaptic 5-HT_3_ receptors mediate fast excitatory synaptic transmission in rat neocortical interneurons, amygdala, and hippocampus, and in ferret visual cortex [45–48]. 5-HT_3_ receptors are also present on presynaptic nerve terminals, where they are thought to mediate or modulate neurotransmitter release [49, 50]. Previously we also have reported that 5-HT_3_ receptor may present presynaptically on TH-neuron in brain stem. However, in the present study, we only examined c-Fos expression in the hypothalamus and brain stem. Therefore, further study also need examine pre- or postsynaptic distributions of 5-HT_3_ receptors under different energy conditions. In addition, Corticosterone and ACTH levels were also examined in fed and fasted mice after treatment with SR 57227 alone or in combination with ondansetron. Corticosterone and ACTH levels were significantly increased by fasting. These findings also confirm our previous report that corticosterone level was significantly enhanced by fasting [28]. However, in the present study, corticosterone and ACTH levels were not affected by SR 57227 alone or in combination with ondansetron.

Taken together, our findings suggest that the inhibitory effect of SR 57227 on feeding behavior in fasted mice may be related to feeding-related neuronal activity (c-Fos) in the hypothalamus and the brain stem, as well as the POMC system in the hypothalamus and the TH system in the brain stem.
